# Slow-release antibacterial film loaded with clove essential oil based on tapioca starch used for bread preservation^☆^

**DOI:** 10.1016/j.fochx.2025.102677

**Published:** 2025-06-20

**Authors:** Hui Chang, Ying Zhao, Jie Zhang, Jian Chen, Tao Yang

**Affiliations:** a School of Pharmacy, International Collaborative Research Center for the Development and Utilization of Tropical Food for Special Medical Purpose, Hainan Medical University, Haikou 571199, China; b Key Laboratory of Food Nutrition and Functional Food of Hainan Province, School of Food Science and Engineering, Hainan University, Haikou 570228, China; c College of Tropical Agriculture and Forestry, Hainan University, Haikou 570228, China

**Keywords:** Green packaging, Starch-based film, Clove essential oil, Slow-release film, Antibacterial, Noncontact preservation

## Abstract

White pollution is an increasingly serious concern. Consequently, the development of green degradable packaging materials has become a research focus. We incorporated different concentrations of clove essential oil (Ceo) (0 %, 0.5 %, 0.75 %, 1.0 %, and 1.25 %, *w/w*) into modified tapioca starch to formulate bacteriostatic compound films. The test results indicated that the barrier properties of the Ceo-series composite films surpassed those of the tapioca starch (TS) film. Specifically, the oxygen permeability of the 1.25 % Ceo film was 7.31 g/m^2^·s, the water vapor permeability decreased to 1.35 g/m^2^·s, and the oil permeability was reduced to 0.15 g mm m^−2^ d^−1^. In addition, the 1.25 % Ceo film exhibited inhibition zones of 4.23 ± 0.28 mm against *Staphylococcus aureus* and 5.02 ± 0.31 mm against *Escherichia coli.* In the sustained release test, the Ceo-series composite films demonstrated a uniform and stable release. Furthermore, their degradability reached approximately 70 % by the 20th day. The 1.25 % Ceo film extended the shelf-life of bread to 9 days, exceeding than that of the commercially available polyethylene (PE) plastic wrap. These results highlight that Ceo-series composite films possess outstanding characteristics as active food packaging materials, particularly for noncontact food preservation.

## Highlights

• Comprehensive studies were carried out on the properties of the Ceo-series films.
• 1.25% Ceo film can prolong the shelf life of bread during storage.
• Composite film can be used as an effective method to realize non-contact preservation.
• Ceo series films have excellent antibacterial and application properties.

## Graphical abstract

**Figure:**
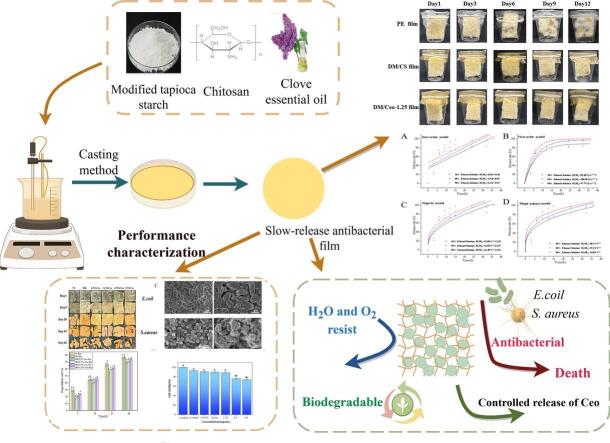

## 1 Introduction

Food spoilage is a worldwide concern. Identifying suitable antibacterial agents and developing novel packaging materials are critical solutions to this problem. To minimize plastic use, researchers have explored biodegradable films derived from natural biopolymers, such as starch, proteins, polysaccharides, and lipids (Istiqomah et al., 2022). Moreover, integrating bioactive compounds, such as peptides, essential oils (EOs), polyphenols, carotenoids, sterols, and vitamins, into biomaterials to form active packaging systems has emerged as a new research avenue that offers functionalities, including antioxidant, antibacterial, and antifungal properties (Kumar et al., 2024).

Among the various natural biopolymers, tapioca starch (TS) stands out due to its strong film-forming ability, high biocompatibility, and broad prospects in edible film preparation (Gutiérrez et al., 2015). However, the practical application of pure TS films is hindered by strong hydrophilicity and poor thermal stability. Current studies have frequently employed modification treatments to alter starch properties (Cheng et al., 2022). In this study, the film matrix was prepared using TS, which was modified via citrate esterification and ultrasonic treatment.

Chitosan (CS), a biodegradable and nontoxic chitin-derived biopolymer with strong film-forming properties is an ideal material for food packaging (Liu et al., 2023). The results of the study by Liu et al. (2009) indicated that due to interactions between the hydroxyl groups of starch and the amino groups of CS, the elongation at break and water vapor transmittance (WVT) of the composite film were significantly enhanced. The study by Pelissari et al. (2009) indicated that the incorporation of CS into starch-based films enhances their mechanical strength, barrier properties, and thermal stability, rendering them appropriate for various food packaging applications. Owing to the considerable potential of CS in film formation, we mixed CS with the modified TS as a film substrate for loading clove essential oil (Ceo).

Essential oils (EOs) have gained attention for their natural origin, effectiveness, and degradability. Previous studies have shown that incorporating EOs into the film matrix could enhance the performance of a composite film (Zhang et al., 2023). For instance, introducing hydrophobic substances, such as EOs, has effectively mitigated high permeability issues of pure starch films (Sun et al., 2020). When an appropriate proportion of EO is added to the film matrix, the film acquires excellent antibacterial properties (Souza et al., 2013). Ceo exhibits excellent antioxidant and antibacterial activities (Lakshmayya et al., 2023). At present, active films containing Ceo are most widely used for meat product storage (Roy et al., 2022). Integrating Ceo into starch films has yielded promising results in the preservation of fruits and vegetables (Liu et al., 2024). However, research on composite films loaded with Ceo for cereal product applications remains limited. To fill this gap, we have provided additional insights in this area of research.

In practical applications, traditional bio-based starch packaging is typically utilized in the form of wrapping (Deng et al., 2024). However, whether this method has adverse effects on food due to the migration of packaging ingredients is still uncertain. Noncontact food preservation refers to the utilization of various technologies that do not require direct contact with the food and are designed to extend shelf life while maintaining quality. This approach is gaining popularity (Fan et al., 2024). To our knowledge, research remains scarce on the preservation effects of starch-based films containing Ceo on cereal products, such as bread, using noncontact treatment methods. Based on previous research, we speculate that tapioca-based active films, modified with Ceo, hold potential for noncontact applications.

Therefore, in this study, we evaluated the properties of these modified tapioca active films containing Ceo, including their release characteristics, antibacterial properties, biodegradability, and biosafety. Additionally, we assessed the impact of the composite films on grain product preservation, particularly for noncontact applications.

## 2 Materials and methods

### 2.1 Materials

TS, Ceo, CS, glycerin, acetic acid, and ethanol were sourced from Macklin Biochemical Co., Ltd. (Shanghai, China). Double-modified TS was synthesized in the laboratory (Chang et al., 2024), the degree of substitution of the dual-modified tapioca starch was 0.231 ± 0.050. Bacteria were sourced from the laboratory, and bread was commercially procured from a local retailer (Ingredients: whole wheat flour, water, yeast and salt).

### 2.2 Preparation of modified starch films

Various film compositions were prepared. The slow-release antibacterial film containing Ceo was prepared as follows: A 2 % CS solution was first prepared by dissolving CS in 1 % (*w*/*v*) acetic acid at 40 °C with constant stirring using a magnetic stirrer for 8 h. A 2 % solution of dual-modified tapioca starch was prepared by heating and stirring the starch at 80 °C for 20 min to ensure complete gelatinization. The CS and DM solutions were combined at a mass ratio of 1:1 (*w*/*w*). Subsequently, glycerin was added to the mixture at a concentration of 20 % of the dry base substance (*w*/*v*, where *v* denotes the total film solution volume).

Ceo was gradually introduced dropwise into the film solution at concentrations of 0 %, 0.5 %, 0.75 %, 1.0 %, and 1.25 % (*w/w*) and thoroughly mixed. The TS film solution was prepared by gelatinizing 2 % raw TS following the previously described procedure, then mixing it with the CS solution at a 1:1 (*w*/*w*) mass ratio. All of the film solutions underwent 40 min of ultrasonication using an ultrasonic machine to ensure complete degassing. The film solution was oven-dried, removed, and carefully peeled off. The films were named as DM film, 0.5 % Ceo film, 0.75 % Ceo film, 1.0 % Ceo film, 1.25 % Ceo film, and TS film. Six parallel samples were prepared for each film.

### 2.3 Rheology

The steady-state and dynamic rheological behaviors of the film fluids were analyzed using Brookfield viscometers (Brookfield models DV-II, USA). The mixture was placed on a 40-mm-diameter sample stage. Subsequently, the starch paste solutions were equilibrated at 25 °C for 120 s. The apparent viscosity (Pa·s) and shear stress (Pa) of the samples were determined at a constant shear stress of 5 Pa, with shear rates ranging from 0.1 to 300 s^−1^ and 300 to 0.1 s^−1^ (Chen, Yan, et al., 2022; Chen, Qin, et al., 2022).

### 2.4 Thermal stability

The thermal stability of various films was analyzed using a TGA analyzer (Q2000, TA, USA). Approximately 5 mg of the composite film was weighed in a crucible. The crucible was heated nitrogen atmosphere from 30 °C to 600 °C, at a heating rate of 10 °C/min, and the weight loss curve was recorded (Chen, Yan, et al., 2022; Chen, Qin, et al., 2022).

### 2.5 Water vapor transmittance (WVP), oxygen permeability (OP), and oil penetration (P_O_)

The WVP was determined using a previously established method (Gao et al., 2024).

(1) WVP=W/t×d/A×Δp

where WVP represents water vapor transmittance (g/pa^−1^·s^−1^·m^−1^); Δ m, the change in weight of the test cup before and after the experiment (g); *t*, the duration of the experiment (h); d, film thickness (mm); A, the film experiment area (m^2^); and Δ p, the pressure difference between the inside and outside of the bottle, which is 3167 Pa.

Oxygen permeability (OP) was determined using the deoxidizer absorption method. The film was uniformly coated on the top of the measuring cup, which had been pre-filled with an appropriate quantity of iron powder. The cup was placed in the dryer, with the temperature held constant at 25 °C for 48 h.

(2) OP=Δm/t×A

where OP represents the oxygen permeability of the composite film (g·m^2^·s); Δ m, the change in the weight of the test cup before and after the experiment (g); *t*, the duration of the experiment (h); and A, the film experiment area (m^2^).

Oil permeability (P_O_) was determined using a previously established method (Song et al., 2024). Soybean oil (6 mL) was placed in six 10-mL centrifuge tubes, and then the tubes were evenly sealed with films. Next, the sample was placed on a filter paper for 48 h.

(3) $$P_{O}=\Delta \;w\times x/A\times t$$

where P_O_ represents the oil permeability (mm·m^−2^·d^−1^); Δ w, the change in weight (g); x, film thickness (mm); A, the effective contact area (m^2^); and t, the storage time (d).

### 2.6 Release of Ceo in different food simulants

Following the experimental methods of Feng et al. (2024), three standard simulants were used to measure the release rate at 4 °C and 25 °C: (1) 10 % (*v*/*v*) ethanol, serving as a nonacidic simulant, (2) 50 % (*v*/*v*) ethanol, serving as a simulant of oil-in-water emulsions and alcoholic foods, and (3) 95 % (*v*/*v*) ethanol, serving as a fatty analog. The entire process lasted over 35 h.

#### 2.6.1 Release kinetic models

Following the research methods of Shi et al. (2024), the release behavior of Ceo in the 1.25 % Ceo film was analyzed in three different types of food-simulating liquids (10 %, 50 %, and 90 % ethanol, representing nonacidic analog, oil-in-water emulsions, and fatty analog, respectively). The release data were modeled using four release kinetic models:

$$\text{Zero}-\text{order}:M_{t}/M_{\infty }=kt,$$

$$\text{First}-\text{order}:M_{t}/M_{\infty }=1-e^{-kt},$$

$$\text{Higuchi}:M_{t}/M_{\infty }=kt^{1/2},$$

$$\text{Ritger}–\text{Peppas}:M_{t}/M_{\infty }=k^{\mathrm{tn}},$$

where M_t_/M_∞_ represents the release percentage Ceo at time, *t*, while *k* and *n* denote the model parameters that describe the release mechanism.

### 2.7 Bacteriostatic performance

#### 2.7.1 Bacteriostatic zone

The bacteriostatic activity of the composite films was assessed using *Escherichia coli* and *Staphylococcus aureus* as model bacterial strains. The films were cut into 10-mm-diameter discs and sterilized for 30 min via ultraviolet (UV) irradiation on a sterile workbench. After applying bacterial solution, the films were placed at the center of a petri dish. The petri dishes were incubated, and the size of the antibacterial zone was measured after 36 h (Maran et al., 2014).

#### 2.7.2 Bacterial membrane integrity

The integrity of bacterial membranes was assessed using a method previously established (Zhou et al., 2024).

#### 2.7.3 Electron microscopy of bacteria

The 1.25 % Ceo film, which exhibited the best antibacterial effect, was selected and co-cultured with bacteria. Its destructive effect on the bacterial cells was further examined using scanning electron microscopy (Thermo Fisher Scientific Brno Co. Ltd.). The bacterial treatment method was adapted from Zhao et al. (2023).

### 2.8 Degradability of composite films

Film samples were cut into 3 × 3 cm pieces, weighed, wrapped in a breathable gauze, buried 5 cm below the soil surface, and monitored for 20 days. Samples were weighed at varying intervals, and the data were recorded (He et al., 2021).

### 2.9 Morphology of degraded composite films

On the 20th day of the degradation experiment, the films were sequentially removed and cleaned, followed by surface morphology observation using a scanning electron microscope (Thermo Fisher Scientific Brno Co., Ltd.).

### 2.10 Film cytotoxicity

The 1.25 % Ceo film (20 mg) was accurately weighed and fully immersed in 1640 medium for 24 h in an incubator at 37 °C. The cytotoxic effects of different films on mouse embryonic fibroblasts (3 T3) were detected via the CCK-8 assay (Yin et al., 2024).

### 2.11 Fresh-keeping experiment of bread

#### 2.11.1 Storage period test

Three types of packaging were designed for evaluating the shelf life of bread: PE packaging, DM film, and 1.25 % Ceo film. The bread slices were cut into rectangles (size: approximately 3 * 2.5 cm). Each sample was placed in a sealed disposable plastic cup with a volume of 150 mL. Images of the bread slices in the three packages were captured on days 1, 3, 6, 9, and day 12 to record the storage status.

#### 2.11.2 Total colony

According to the method described by Srivastava et al., the colonies in the bread on different preservation days was determined (Srivastava et al., 2024).

#### 2.11.3 Low-field nuclear magnetic resonance (LF-NMR) analysis

Changes in the water status and distribution characteristics of bread on different days were detected using an LF-NMR instrument (model). The spin–spin relaxation time constant was calculated. Magnetic resonance imaging (MRI) was employed to process the images and obtain the pseudo-color map (Geeta et al., 2024).

#### 2.11.4 Sensory evaluation of bread samples

The sensory attributes evaluated were color, texture, and appearance. Panel evaluation was performed by a panel consisting of 10 trained members. All panelists had completed 120 h of training in all aspects of sensory techniques and analysis. In addition, they had accumulated approximately 100 h of practical testing experience across a diverse array of food products. Finally, sensory properties were scored in the final scale from 1 to 100 (Vicente et al., 2024).

### 2.12 Statistical analysis

All data were analyzed using Origin 2022 and SPSS, with a *p* < 0.05 significance level to determine statistical significance.

## 3 Results and discussion

### 3.1 Rheological analysis

**Fig. 1:**
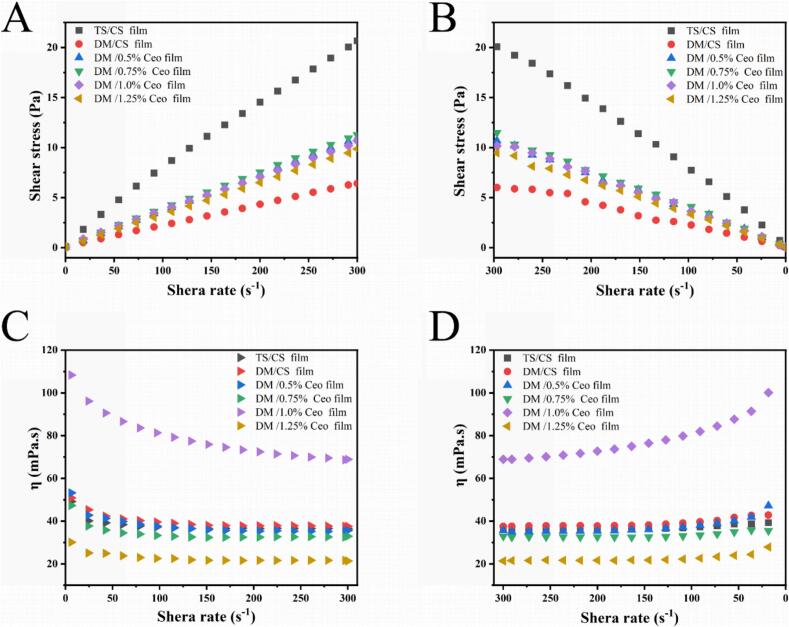
Rheological properties of different film solutions. (A) Shear stress as a function of shear rate (0–300 s^−1^); (B) shear stress as a function of shear rate (300–0 s^−1^); (C) apparent viscosity as a function of shear rate (0–300 s^−1^); (D) apparent viscosity as a function of shear rate (300–0 s^−1^).

The shear stress (Pa) and apparent viscosity (Pa·s) of different film solutions were measured through dynamic rheological analysis (Fig. 1) (Oyeoka et al., 2021). Fig. 1A and B illustrate the shear stress and shear rate results of the various composite films. The shear stress (Pa) of the DM film solution was markedly lower than that of the TS film solution. Increasing Ceo concentration led to gradual reduction of the shear stress (Pa) of the film solutions.

The apparent viscosity (Pa·s) of all the film fluids declined with increasing shear rate, exhibiting shear-thinning behavior (Fig. 1C and Fig. 1D).The results indicated that all the samples were pseudoplastic fluids (non-Newtonian fluids) (Lu et al., 2024). Additionally, the modified DM film solution exhibited notably lower viscosity than the TS film solution system. The viscosity reduction likely stems from starch intermolecular structure disruption, weakened intermolecular binding forces, and reduced entanglement, thereby decreasing the viscous flow resistance (Zhou et al., 2021). In Fig. 1D, the addition of Ceo can significantly reduce the apparent viscosity of the film solution system, with higher concentrations resulting in greater decreases. These results indicate that the Ceo-based composite films, due to their lower shear stress and apparent viscosity, exhibit superior flowability during film-forming processes such as casting and spraying (Mileti et al., 2024).

### 3.2 TGA analysis

**Fig. 2:**
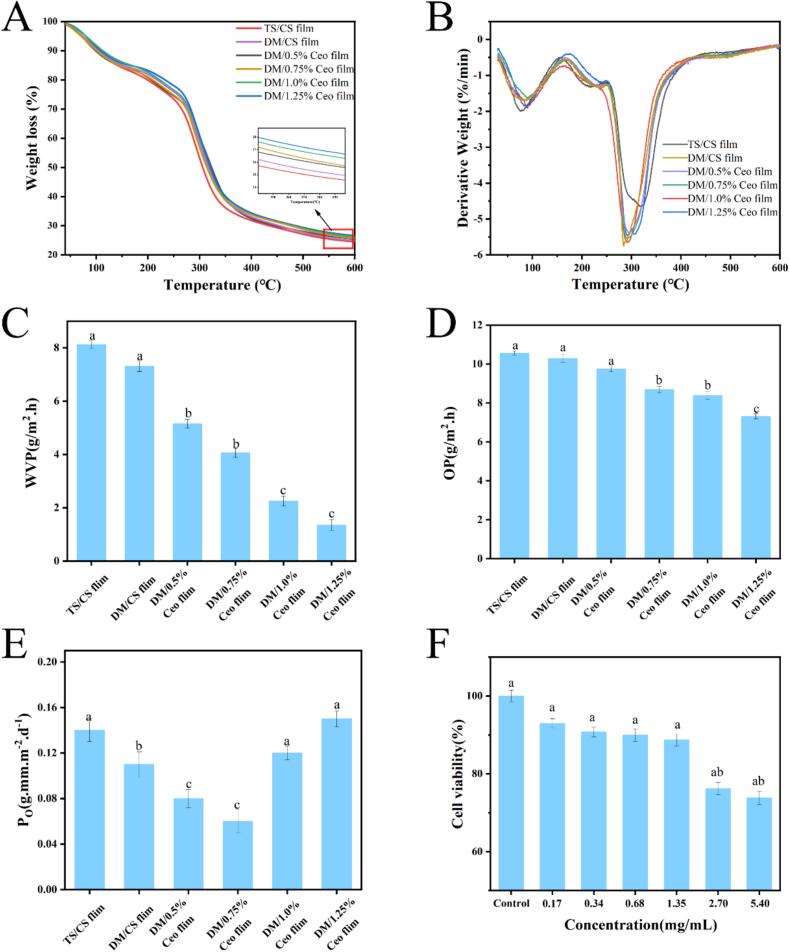
(A) Thermogravimetric analysis (TGA); (B) derivative thermogravimetry (DTG); (C) water vapor permeability (WVP) of composite films; (D) oxygen permeability (OP) of composite films; (E) oil permeability (P_O_) of composite films; (F) cytotoxicity evaluation of the 1.25 % CeO composite film. Vertical bars represent the standard deviation (mean ± SD, *n* = 3). Different lowercase letters indicate statistically significant differences (*P* < 0.05).

As shown in Fig. 2A, at 550 °C, the DM film lost 74.534 % of its weight, while the DM/1.25 % Ceo film exhibited a weight loss of 72.305 %, indicating improved thermal stability due to Ceo addition. This phenomenon could be attributed to the promotion of the interaction between CS and starch polymer molecules by Ceo, which increased the compactness of the network structure of the film and impeded small molecule volatilization (Yu et al., 2023).

Fig. 2B illustrates that all samples underwent a three-step decomposition, exhibiting largely similar thermogravimetric behavior across different film components. Relative to the TS film, the initial decomposition temperature of the modified DM film increased from 74.75 °C to 86.03 °C, indicating notable enhancement in thermal stability. Additionally, the first decomposition stage of the Ceo-containing composite film formed a distinct peak below 100 °C in the DTG curve (T_max_ ≈ 84.88 °C–94.18 °C), attributed to free water evaporation within the film matrix (Yu et al., 2024).

The enhanced thermal stability of the composite film may result from interactions between Ceo and the film matrix. The phenolic hydroxyl group (−OH) of eugenol can form hydrogen bonds with the amino groups (−NH₂) of CS and the hydroxyl groups (−OH) of TS (Song et al., 2025). Hydrogen bonds, which are strong noncovalent interactions, can increase intermolecular binding forces. Additionally, studies have demonstrated that the addition of clove oil increases the degree of crosslinking in the film, thereby improving its thermal stability (Zhang et al., 2025). These results suggest that the Ceo-based composite films are suitable for food packaging that requires thermal treatment or storage at high temperatures, owing to their superior thermal stability and structural integrity under elevated temperature conditions (Santhosh et al., 2024).

### 3.3 Analysis of WVP, OP, and P_O_

Figs. 2C–E illustrate the experimental results of WVP, OP, and P_O_ of different films. As depicted in Fig. 2C, increasing Ceo film concentration reduced WVP from 5.15 to 1.35 g/m^−1^·s^−1^·P^−1^ (0.5 %–1.25 % Ceo film). This finding was consistent with the results reported by Mulla et al., which showed that incorporating thyme essential oil (TEO) in biopolymer-based films significantly reduced WVP, likely due to the hydrophobic nature of TEO (Mulla et al., 2024). These findings suggest that hydrophobic EOs effectively suppress water migration, enhancing the water resistance of composite films.

As illustrated in Fig. 2D, the OP value decreased from 9.75 to 7.31 g/m^2^·s (0.5 %–1.25 % Ceo film). The uniform distribution of droplets in the Ceo-loaded composite film likely disrupts the hydrophilic phase continuity of the film-forming matrix, hindering the migration of water molecules. Additionally, emulsion drops may limit polymer molecular chain mobility by filling the void in the film-forming matrix, thereby augmenting the gas barrier property of the composite film (Gao et al., 2024).

Relative to the DM film, the P_O_ value of the 0.5 % Ceo film decreased from 0.11 to 0.08 g mm m^−2^ d^−1^ (Fig. 2E). However, as the Ceo concentration in the film increased, the P_O_ values of the 0.75 %, 1.0 %, and 1.25 % Ceo films did not decrease further. Instead, they rose from 0.06 g mm m^−2^ d^−1^ to 0.12 and 0.15 g mm m^−2^ d^−1^, respectively. A lower Ceo concentration (0.5 %–0.75 %) slows the oil penetration rate, effectively reducing the oil permeability of the composite film. In contrast, when the Ceo concentration exceeds 0.75 %, the film matrix becomes saturated with EO, leading to an increase in the P_O_ value (Chen et al., 2024).

### 3.4 Release of Ceo

A UV spectrophotometer was used to scan Ceo in the full band (Fig. S1). The results indicated that Ceo exhibits maximum absorbance at 283 nm. The standard equation was y = 19.714 x + 0.031, R^2^ = 0.9981 (Fig. S2).

**Fig. 3:**
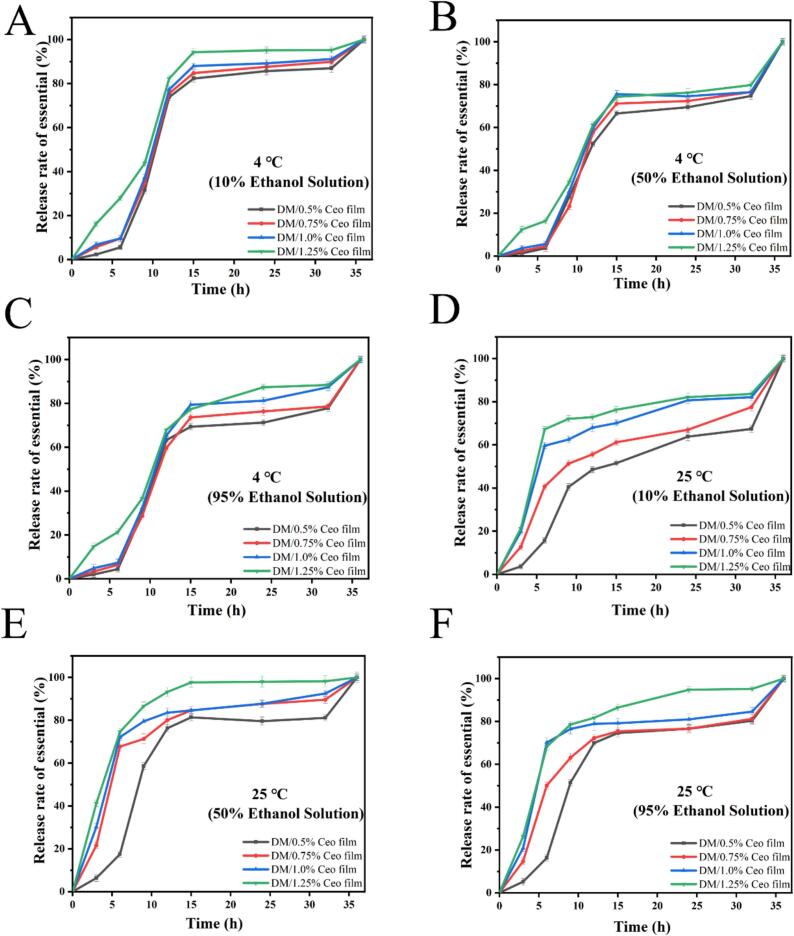
Release of composite films in different simulated liquids. (A) In nonacidic food simulation solution (10 % ethanol, solution) at 4 °C; (B) in oil-in-water solution food simulation solution (50 % ethanol solution) at 4 °C; (C) in fatty food simulation solution (95 % ethanol solution) at 4 °C; (D) in nonacidic food simulation solution (10 % ethanol solution) at 25 °C; (E) in oil-in-water solution and alcoholic food simulation solution (50 % ethanol solution) at 25 °C; (F) in fatty food simulation solution (95 % ethanol solution) at 25 °C. Vertical bars represent the standard deviation of the mean (n = 3).

Fig. 3A–F compares the EO release rates from the Ceo-series composite films in various food simulants across different temperatures. At 4 °C, EO release was the fastest in the nonacidic food (10 % [*v*/*v*] ethanol simulant) and the slowest in the oil-in-water food (95 % [*v*/*v*] ethanol simulant). For fatty foods (simulated with 50 % [*v*/*v*] ethanol), Ceo release occurred at an intermediate rate, consistent with the previous finding (Cui et al., 2021). At 25 °C, the EO release was highest in the fatty food ethanol simulant (50 % [*v*/*v*] ethanol), lowest in the nonacidic food ethanol simulant (10 % [*v*/*v*] ethanol), and intermediate in the oil-in-water food ethanol simulant (95 % [*v*/*v*] ethanol). These findings suggest that the composite film series is better suited for fatty food preservation at 25 °C.

EO release rates from composite films with varying concentration gradients initially surged before declining, likely due to unincorporated Ceo on the film surface, causing rapid during early testing (Kamwilaisak et al., 2022). Additionally, rapid release of Ceo from the composite films primarily occurred within the first 15 h. Subsequently, the release rate stabilized at a slower pace. After 15 h, ethanol began to interact with the Ceo loaded in the matrix through the interstices of the composite film, gradually compromising the integrity of the film and leading to a slower Ceo release. This indirect release mechanism resulted in a more uniform and sustained release profile compared with the direct release of EO (Feng et al., 2024).

#### 3.4.1 Analysis of release kinetic models

The release kinetics of EO from the 1.25 % Ceo film were analyzed using four kinetic models: the first-order Hixson–Crowell, Higuchi, Korsmeyer–Peppas, and Baker–Lonsdale models. The results are presented in Fig. S3, Fig. S4, and Table S1. As presented in Figs. S3 and S4, in the Ritger–Peppas model, the release exponents (n) of Ceo from the 1.25 % Ceo film in three different simulated solutions were 0.52, 0.61, and 0.65 at 4 °C, respectively. These values lie within the range of 0.45 to 0.89, suggesting that the release mechanism conforms to anomalous transport (non-Fickian diffusion) (Yang et al., 2024). At 25 °C, the release exponent (n) was less than 0.45 in all three simulated solutions, indicating Fickian diffusion behavior (Bansal et al., 2025).

As indicated by the R^2^ values of all kinetic models presented in Table S1, the first-order model provided the best fit for the release data of Ceo from the 1.25 % Ceo film in three food simulants at different temperatures (4 °C and 25 °C) (Ye et al., 2025). Therefore, it is inferred that the first-order model may act as a promising predictor of Ceo release rates from the 1.25 % Ceo film in various food matrices.

### 3.5 Bacteriostatic performance

#### 3.5.1 Bacteriostatic zone

**Fig. 4:**
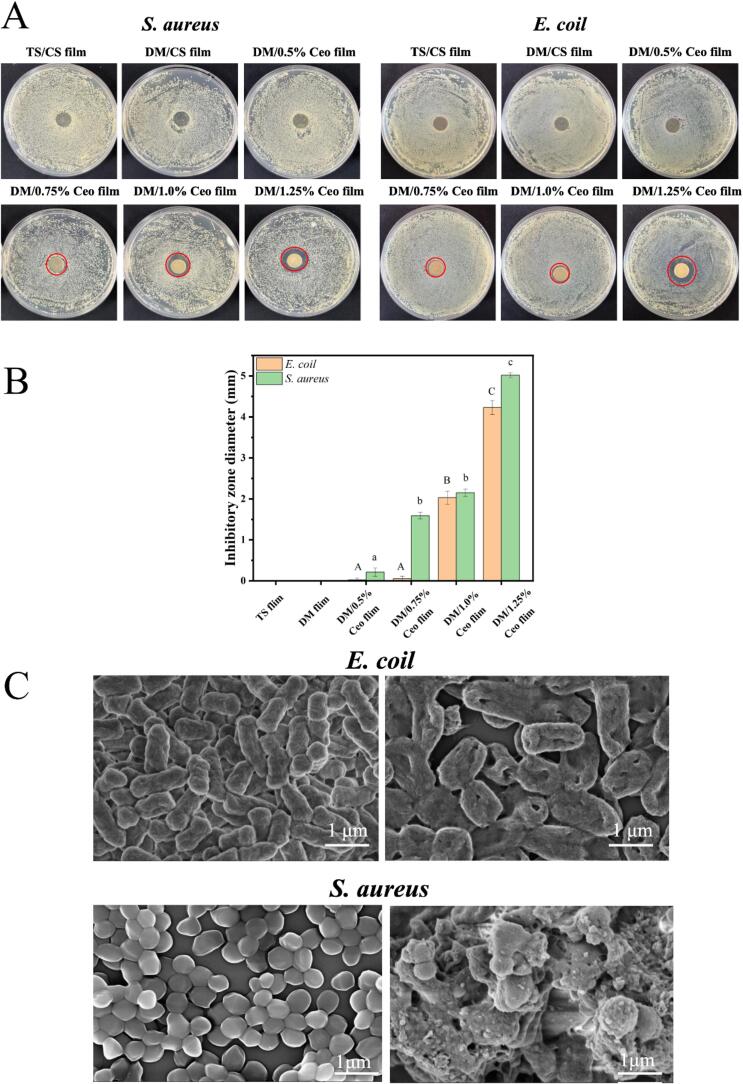
(A) Bacteriostatic zone; (B) diameter of the bacteriostatic zone (mm); (C) scanning electron microscopy of bacteria (scale bars: 1 μm). Vertical bars represent the standard deviation of the mean (n = 3); different letters represent significant differences (*P* < 0.05).

The antibacterial activities of different films against two foodborne pathogens (*S. aureus* and *E. coli*) were tested. The antibacterial zone sizes of composite films were measured using the agar diffusion method (Fig. 4A). In *S. aureus*, the smallest inhibition zone was 0 ± 0.12 mm (DM film). In contrast, the largest inhibition zone was 4.23 ± 0.28 mm (1.25 % Ceo film). In *E. coli*, the smallest zone was 0 ± 0.09 mm (TS film), whereas the largest zone was 5.02 ± 0.31 mm (1.25 % Ceo film) (Fig. 4B).

The TS and DM films exerted negligible inhibitory effects on the growth of both bacterial strains. The inhibitory effect of the Ceo-series films on bacterial growth tended to increase with the increase in the Ceo concentration. Ceo exerts antibacterial effects as eugenol destroys the bacterial cell membrane, thereby causing bacterial death (Chang et al., 2023). In addition, the inhibitory effect of Ceo on gram-positive bacteria (*S. aureus*) was relatively strong (Xing et al., 2024).

#### 3.5.2 Integrity of bacterial cell films

Nucleic acids have a maximum UV absorption value of 260 nm, and the absorption value is proportional to the nucleic acid concentration (Istiqomah et al., 2022). Thus, the degree of damage to the bacterial cell membrane was analyzed through the measurement of the optical density at 260 nm, and the integrity of the bacterial cell membrane is inferred. The results are presented in Table 1. After *S. aureus* and *E. coli* were treated with the gradient Ceo films, the absorbance values of those films at 260 nm were significantly higher than those of the DM film and finally reached 3.654 and 3.741 in the 1.25 % Ceo film and nucleic acid leaked was 66.5 and 79.3 μL, respectively. Ceo exerted a certain destructive effect on the cell film in both bacteria. The degree of damage caused by Ceo depended on its concentration; the higher the Ceo concentration, the greater the degree of damage.

**Table 1:** Nucleic acid leakage of *Escherichia coli* and *Staphylococcus aureus* in different composite films.

Samples	Nucleic acid content *E. coli* (A_260nm_)	Nucleic acid content *S. aureus* (A_260nm_)	Protein content (μL) *E. coli*	Protein content (μL) *S. aureus*
TS film	0.765	0.775	12.7	10.2
DM film	0.614	0.618	5.5	6.3
DM/0.5 % Ceo film	0.942	1.174	13.9	19.6
DM/0.75 % Ceo film	1.268	1.294	36.5	46.1
DM/1.0 % Ceo film	2.528	2.117	44.7	69.1
DM/1.25 % Ceo film	3.654	3.741	66.5	79.3

Previous studies have shown that the antibacterial efficacy of Ceo is primarily attributed to its major constituent, namely, eugenol (Zeng et al., 2025). Eugenol can disrupt the structural integrity of bacterial cell membranes and markedly increase their permeability. This disruption facilitates the leakage of intracellular macromolecules, such as proteins, nucleic acids, and DNA, into the extracellular environment (Silva, de Lima, et al., 2024; Silva, Neto, et al., 2024). Consequently, intracellular homeostasis is severely compromised, ultimately leading to bacterial cell death. Furthermore, eugenol can further modulate the permeability of the cell membrane, causing efflux of essential intracellular substances, including ATP and nucleic acids. This process effectively interferes with the normal metabolic pathways of bacteria, exerting a pronounced inhibitory effect on their growth and proliferation.

#### 3.5.3 Electron microscopy of bacteria

SEM images (Fig. 4C) showed that the bacteria that were not co-cultured with the Ceo composite films appeared intact. After co-culture with the 1.25 % Ceo film, the morphology of the bacteria changed. Specifically, *E. coli* was deformed, and its rod became wider and perforated in the middle, causing its contents to overflow. *S. aureus* lost its smooth spherical shape, generated numerous folds on its surface, and demonstrated severe collapse. In addition, the bacterial morphology was no longer intact, and some viscous substances were produced (Ruiz-Navajas et al., 2013).

### 3.6 Degradation experiment results

**Fig. 5:**
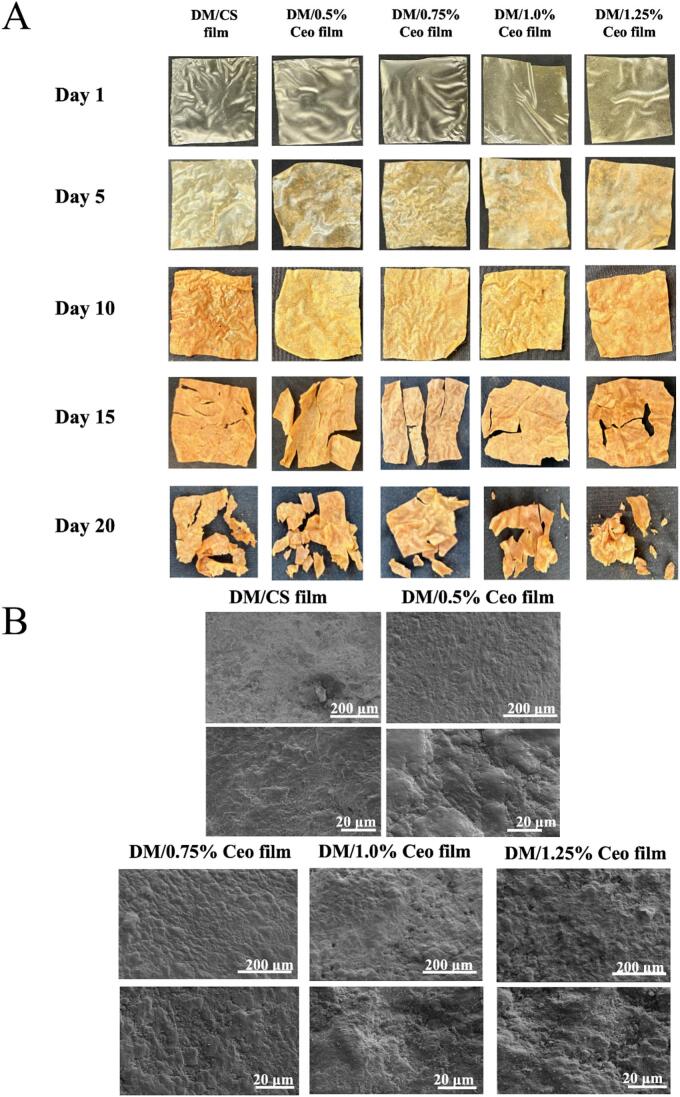
(A) Morphological changes of the composite films during the 20-day degradation process; (B) scanning electron microscopic images of the composite films after 20-day degradation (scale bars: 200 μm, 20 μm). Vertical bars represent the standard deviation of the mean (n = 3); different letters represent significant differences (*P* < 0.05).

Fig. 5 presents the degradation condition of the composite films. The surface of different composite films crumpled and curled on day 5 of the experiment, which may have been caused by soil–water absorption by the composite films and indicated the beginning of degradation (Fig. 5A). In Fig. 5B, porous structures of 1.25 % Ceo film were observed on the film surface. These structures can facilitate water absorption, swelling, and microbial degradation, thereby accelerating the disintegration rate (Zhao et al., 2025). Moreover, soil microorganisms utilize TS in composite films as the sole carbon source to sustain their metabolic activities, thereby providing suitable conditions for microbial metabolism, which is the main reason for film degradation (Wicaksono et al., 2022). On the 20th day of the degradation experiment, compared with the images on day 0, the smooth surface of the composite film had completely disappeared as degradation progressed. This finding suggests that the sample was affected by microorganisms, with significant erosion of the surface. The internal structure of the composite film was fully exposed after 20 days of biodegradation.

As presented in Fig. S5, the degradation rates of the DM film and Ceo-series composite films after 20 days reached 73.5 % (DM film), 69.8 % (0.5 % Ceo film), 70.2 % (0.75 % Ceo film), 71.3 % (1.0 % Ceo film), and 77.4 % (1.25 % Ceo film). The degradation rates of the Ceo-series composite films increased as the Ceo concentration gradient increased. Previous studies have demonstrated that the soil degradation rates of PP and PE were extremely low, with a weight loss of less than 2 % in all groups within a 1-year observation period, virtually indicating no degradation (Li et al., 2025). This is attributed to the high hydrophobicity of PP and PE owing to their long carbon chains, which prevent them from interacting with soil moisture to undergo degradation. In our study, the degradation rate of the 1.25 % Ceo film reached 77.4 % on day 20, which was 38.7 times greater than the maximum degradation rates of PP and PE (Bersaneti et al., 2021). In summary, the Ceo-based composite films exhibited remarkable degradability, making them a promising candidate for sustainable packaging applications.

### 3.7 Ceo film cytotoxicity

The cytocompatibility of the 1.25 % Ceo film was tested via the CCK-8 assay (Fig. 2F). The cell survival rate exceeded 73 % at the concentration range of 0.17–5.40 mg/mL, indicating that the film was nontoxic to 3 T3 cells and thus could be safely used (Silva, de Lima, et al., 2024; Silva, Neto, et al., 2024).

### 3.8 Bread preservation experiment results

**Fig. 6:**
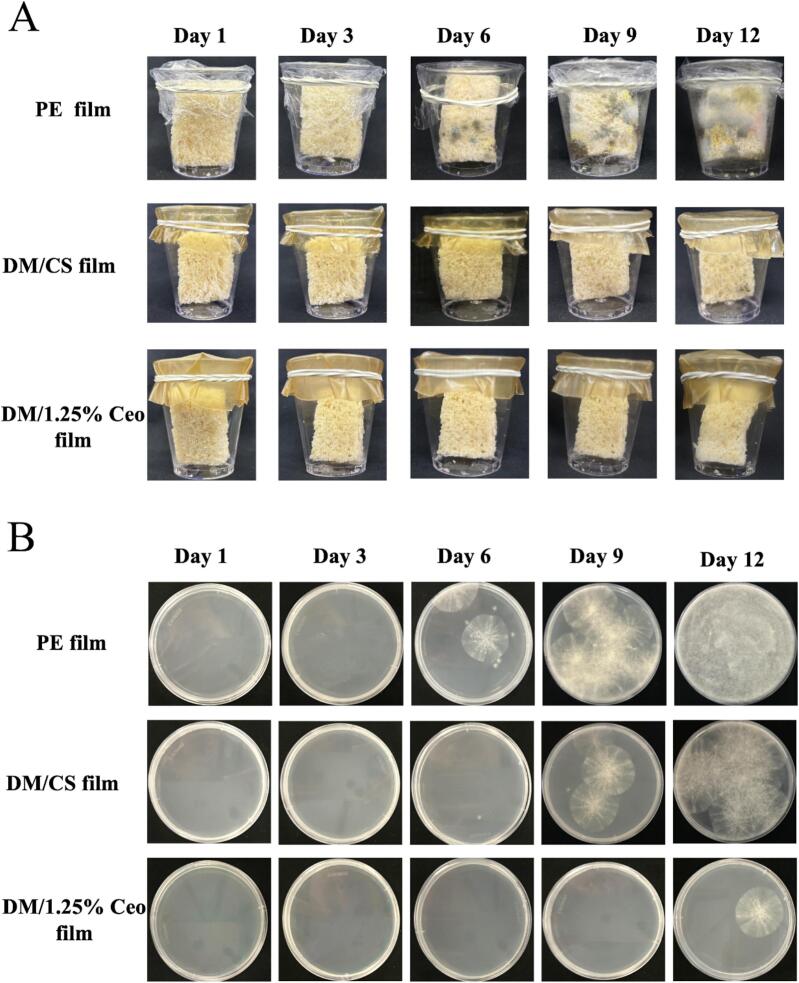
(A) The visual appearance changes of bread with different treatments during 12 days of storage; (B) total number of colonies in bread during the 12-day preservation process.

The preservation effect of the 1.25 % Ceo film was evaluated using a noncontact method with commercially available PE and DM films as controls (Fig. 6A and Fig. 6B). In traditional composite films, food preservation is generally handled through direct contact. However, the starch composite film matrix contains numerous carbohydrates, which may inadvertently provide additional nutrients for microorganisms, making microbial inhibition less effective (Srivastava et al., 2024). The fresh-keeping experiment of bread was conducted under noncontact conditions (Fig. 6A). When the PE film was used, the mold began propagating on the bread on day 6 (Fig. 6B). Similarly, in the DM film experimental group, the mold began appearing on day 6, but it was not apparent. In the 1.25 % Ceo film experimental group, the shelf life of the bread was extended to 9 days, and no visible mold colony was observed on the surface, which indicated that this 1.25 % Ceo film is promising as a food packaging material.

### 3.9 Low-field nuclear magnetic resonance (LF-NMR) results

**Fig. 7:**
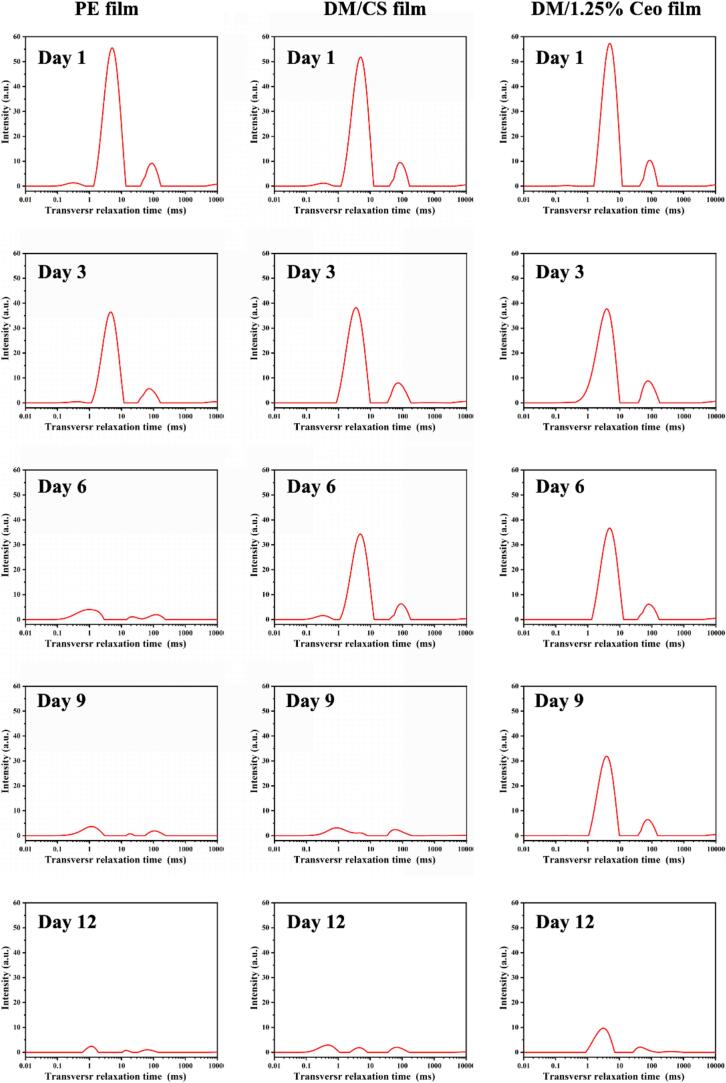
Changes in moisture content of bread packaged with different composite films (PE film, DM film and 1.25 % Ceo film) during 12-day storage.

The area under the curve of the LF-NMR relaxation assay reflects the water content in the food. The transverse relaxation time (Fig. 7) serves as a measure of water mobility, with the area under the curve indicating the water content and its distribution in the food matrix. The peak area was used to represent the water content in the bread, with changes in the peak area strongly associated with the water-holding capacity of bread (Zhang et al., 2020). Specifically, T_21_ (0.1–1 ms), T_22_ (4–15 ms), and T_23_ (40–110 ms) corresponded to bound, immobilized, and free water, respectively (Łopusiewicz et al., 2023).

Fig. 7 (left: PE film; middle: DM film; right: 1.25 % Ceo film) presents a trend of water retention in the three experimental groups after the storage time extension. On day 1, all three groups of bread samples presented the highest level of bound water, with similar peak areas across the samples. By day 3, the water retention capacity began declining in all the groups, and by day 6, the PE group presented the most significant moisture loss, whereas the DM film and the 1.25 % Ceo film groups remained relatively stable. By day 9, the 1.25 % Ceo film group showed superior moisture retention compared with the PE and DM film groups. This result indicated that the 1.25 % Ceo film group exhibited a relatively good water retention effect.

Luna-Niño et al. (2025) used a coating of chayote root starch/cinnamon EO/glycerol composite film for bread preservation and reported that on day 4 of storage, the hardness of bread in the experimental group was 5.1 times greater than that in the control group. The negative impact on bread hardness was likely due to the accelerated moisture loss and migration induced by the drying process of the coating solution. In our study, the bread sample treated with the 1.25 % Ceo film demonstrated favorable moisture retention on day 9 of storage, suggesting that the noncontact preservation method has great potential.

MRI techniques were employed to reflect water distribution and migration within the bread (Mittal et al., 2023). The pseudo-color map (Fig. S6) showed that the water content in the bread gradually decreased, which was related to the aging of the bread (Fan et al., 2024). In the PE film, yellow hydrogen protons basically disappeared on day 9, whereas black appeared in the pseudo-color image on day 12, indicating severe water loss of bread. In the 1.25 % Ceo film, the presence of yellow hydrogen protons was still observed on day 12, which indicated that the water retention of the bread was relatively good under these conditions. This could be related to the lower water vapor permeability of the film containing Ceo. These results confirmed that the composite films treated using the noncontact method exhibited excellent water retention.

### 3.10 Analysis of sensory evaluation of bread samples

Fig. S7 illustrates the sensory radar images of the bread on days 1, 6, and 12, which were used to evaluate the color, texture, and appearance of the bread (Protonotariou et al., 2020). On day 1, no significant differences were observed in the scores for color, texture, and appearance among the three treatment groups. On day 6, mold was observed on the bread in the PE group (Fig. 6B), with the appearance score decreasing from 95 to 40. By day 12, all three bread groups had become moldy and spoiled. However, compared with the other groups, the bread in the 1.25 % Ceo film group exhibited relatively less severe quality deterioration, which could be attributed to the antimicrobial properties of the Ceo released by the film. Overall, 1.25 % Ceo film showed potential as a food packaging material.

## 4 Conclusion

This study primarily examined the performance of Ceo-series composite films. Rheological test results indicate that composite films fluids belonged to pseudo-shaping fluids and formed a stable network structure with superior elasticity. In the antibacterial experiment, the 1.25 % Ceo film exerted the best inhibitory effect on two foodborne pathogens and exhibited great potential as an antibacterial packaging material. Protein leakage from both pathogens was attributed to Ceo action, with the effect intensifying as Ceo concentration increased. EO release experiments suggest that Ceo-series composite films are better suited for preserving nonacidic foods at 4 °C and alcoholic foods at 25 °C. Finally, the 1.25 % Ceo film was chosen for studying the biosafety and bread preservation studies. This film demonstrated excellent biocompatibility and markedly extended the shelf life of bread. LF-NMR results confirmed the film's water retention capability. In summary, the 1.25 % Ceo film is a promising candidate for green antibacterial packaging. Future research will include comprehensive studies and sensory evaluations of other perishable foods.

## CRediT authorship contribution statement

**Hui Chang:** Conceptualization, Data curation, Writing - original draft; **Ying Zhao:** Validation; **Jie Zhang:** Project support; **Jian Chen:** Writing - review & editing; **Tao Yang:** Software, Writing - review & editing, Funding acquisition.

## Declaration of competing interest

The authors declare that they have no known competing financial interests or personal relationships that could have appeared to influence the work reported in this paper.

## Data Availability

No data was used for the research described in the article.
