# Supplementary material for: Slow-release antibacterial film loaded with clove essential oil based on tapioca starch used for bread preservation
Source: Food Chem X. 2025 Jun 20;29:102677. doi: 10.1016/j.fochx.2025.102677 (PMC12268568; doi:10.1016/j.fochx.2025.102677)
Supplement: Supplementary material [file mmc1.docx]

**Slow-release antibacterial film loaded with clove essential oil based on tapioca starch used for bread preservation**

Hui Chang ^a, b^, Ying Zhao ^c^, Jie Zhang ^c^, Jian Chen ^b*^, Tao Yang ^a*^

^a^ School of Pharmacy, International Collaborative Research Center for the Development and Utilization of Tropical Food for Special Medical Purpose, Hainan Medical University, Haikou 571199, China

^b^ Key Laboratory of Food Nutrition and Functional Food of Hainan Province, School of Food Science and Engineering, Hainan University, Haikou 570228, China

^c^ College of Tropical Agriculture and Forestry, Hainan University, Haikou, 570228, China

Corresponding authors:

E-mail addresses: <chenjian19850702@163.com> (J. Chen), [taoyangfood@muhn.edu.cn](mailto:taoyangfood@muhn.edu.cn) (T. Yang).

**Fig. S1** Full wavelength scanning of Ceo.

**Fig. S2** Standard curve of Ceo.

**Fig. S3** Release kinetics of Ceo from the 1.25% Ceo film in different food matrices at 4 °C, including 10% ethanol, 50% ethanol, and 95% ethanol. (A) Zero-order model; (B) First-order model; (C) Higuchi model; (D) Ritger-Peppas model.

**Fig. S4** Release kinetics of Ceo from the 1.25% Ceo film in different food matrices at 25 °C, including 10% ethanol, 50% ethanol, and 95% ethanol. (A) Zero-order model; (B) First-order model; (C) Higuchi model; (D) Ritger-Peppas model.

**Fig. S5** Degradability of the 0.5–1.0% Ceo series composite films.

**Fig. S6** Spatial distribution of water content in bread under different packaging conditions visualized by false-color mapping (12-day storage period).

**Fig. S7** Sensory evaluation results of bread under different packaging conditions and storage periods. (A) 1 day; (B) 6 days; (C) 12 days.

**Fig. S1**


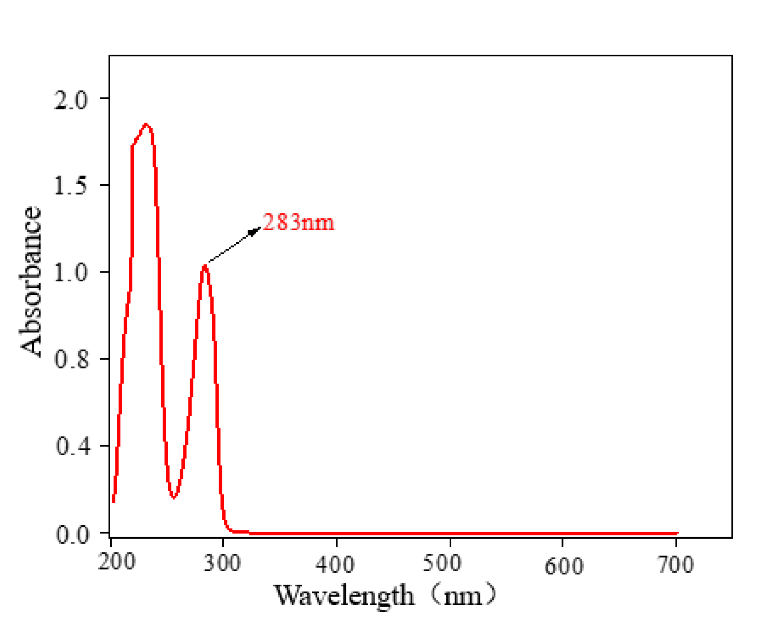


**Fig. S2**


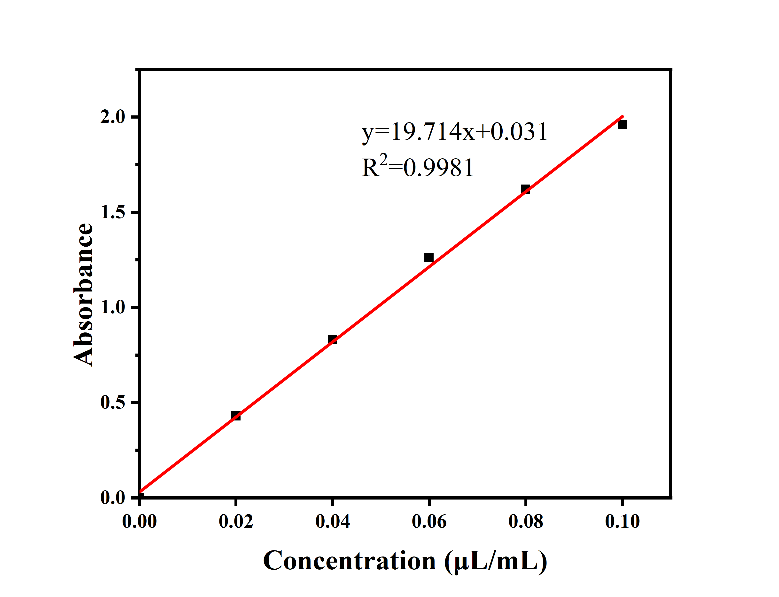


**Fig. S3**


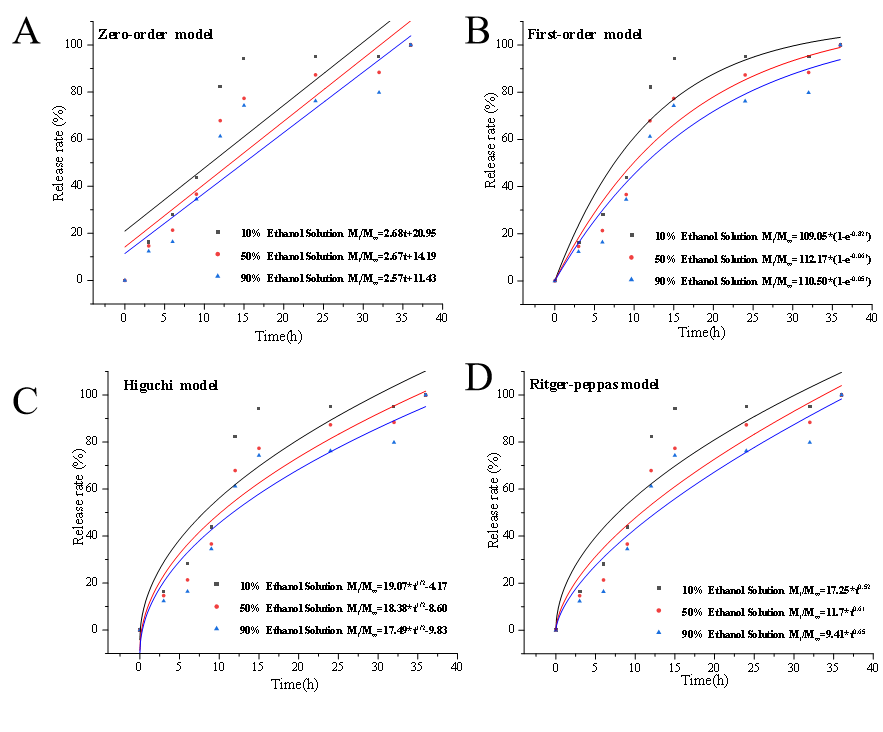


**Fig. S4**


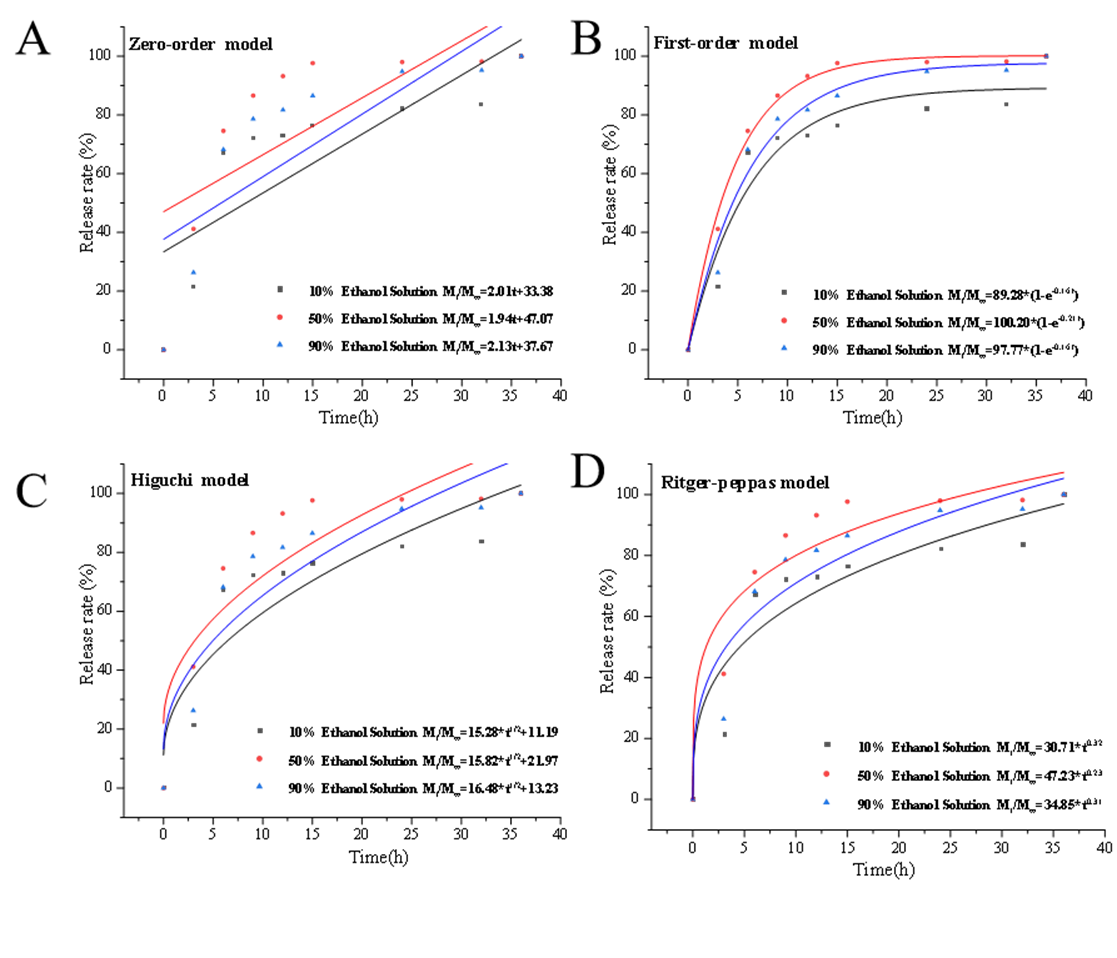


**Fig. S5**


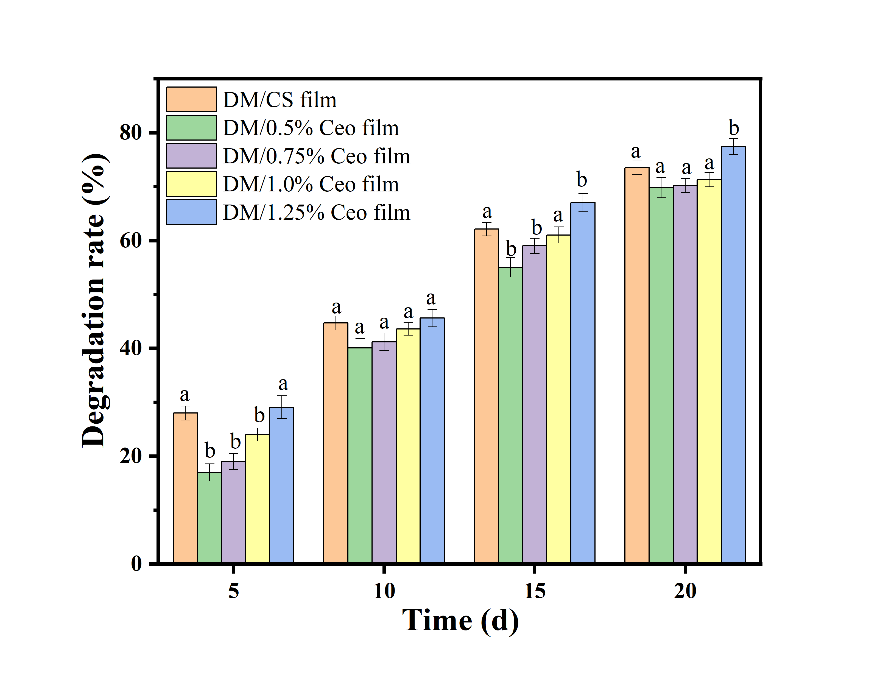


**Fig. S6**


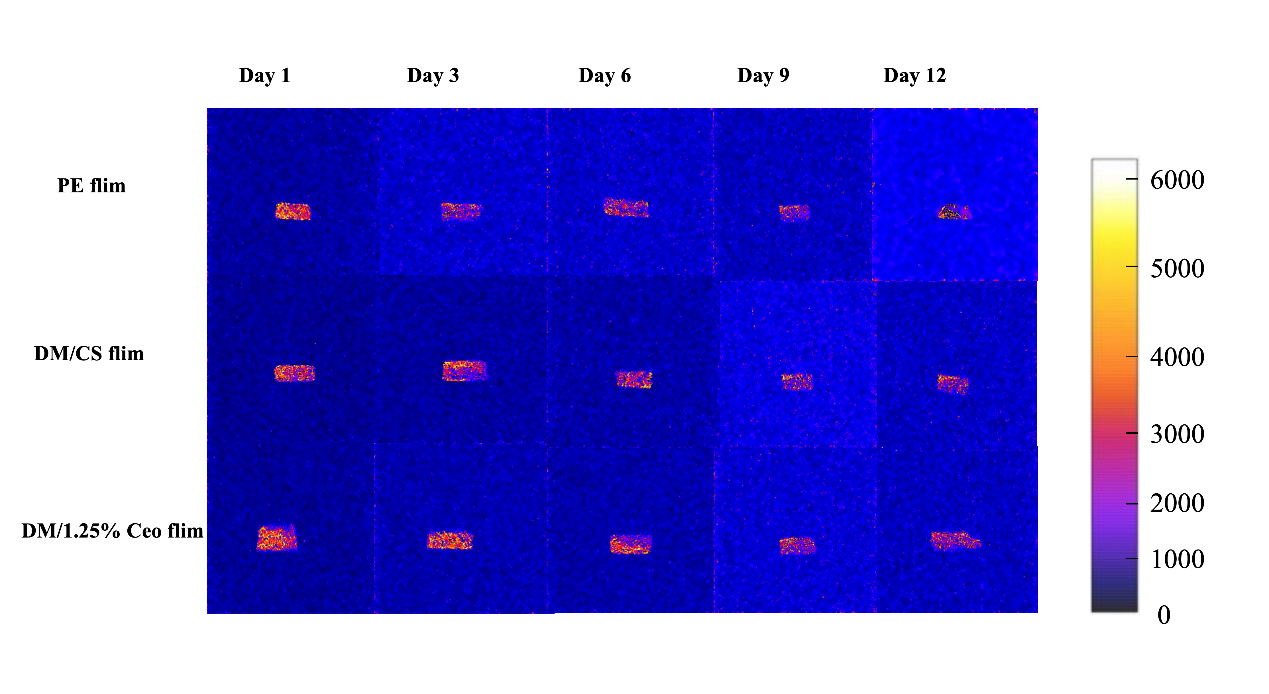


**Fig. S7**


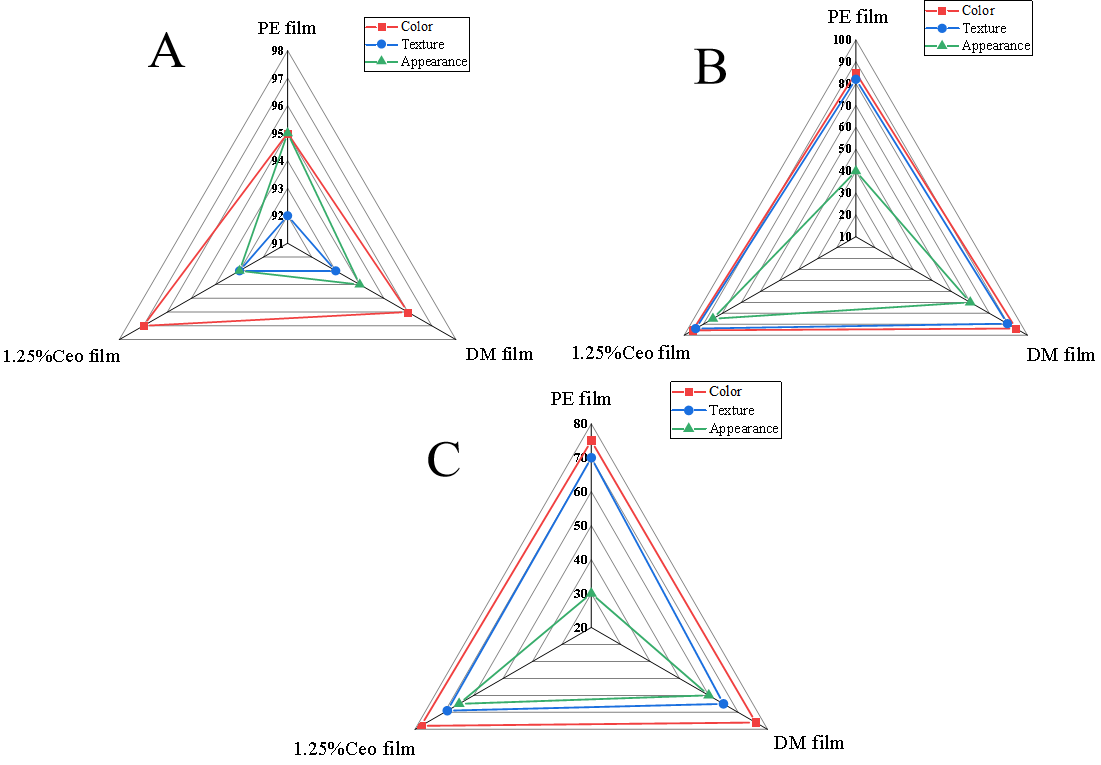


**Table S1**

The correlation coefficients (R^2^) for different models used to describe the release kinetics of Ceo in different food simulation solution at 4 ℃ and 25 ℃.

| Temperature | Simulated liquid | Model | R^2^ | Simulated liquid | Model | R^2^ | Simulated liquid | Model | R^2^ |
| --- | --- | --- | --- | --- | --- | --- | --- | --- | --- |
| 4 ℃ | 10% Ethanol | Zero-order | 0.74 | 50% Ethanol | Zero-order | 0.85 | 90% Ethanol | Zero-order | 0.86 |
|  |  | **First-order** | **0.92** |  | **First-order** | **0.95** |  | **First-order** | **0.93** |
|  |  | Higuchi | 0.86 |  | Higuchi | 0.91 |  | Higuchi | 0.90 |
|  |  | Ritger-peppas | 0.86 |  | Ritger-peppas | 0.92 |  | Ritger-peppas | 0.91 |
| 25 ℃ | 10% Ethanol | Zero-order | 0.64 | 50% Ethanol | Zero-order | 0.52 | 90% Ethanol | Zero-order | 0.63 |
|  |  | **First-order** | **0.94** |  | **First-order** | **0.99** |  | **First-order** | **0.98** |
|  |  | Higuchi | 0.87 |  | Higuchi | 0.79 |  | Higuchi | 0.85 |
|  |  | Ritger-peppas | 0.89 |  | Ritger-peppas | 0.92 |  | Ritger-peppas | 0.91 |
